# Dairy- and Milk-Inspection1Paper read before the Pennsylvania State Veterinary Medical Society, September 21, 1897.

**Published:** 1897-12

**Authors:** C. Courtney McLean

**Affiliations:** Meadville, Pa.


### THE JOURNAL
OF
### COMPARATIVE MEDICINE AND VETERINARY ARCHIVES.

VOL. XVIII. *DECEMBER, 1897.* No. 12.

### DAIRY- AND MILK-INSPECTION.[1]

BY C. COURTNEY McLEAN, V.S.,
MEADVILLE, PA.

PURE milk is a perfect food, and one of the most important of animal origin, as it contains all the different properties arranged in the proportions best suited for nutritive purposes, and may be regarded as the typical food.

Impure milk is a dangerous poison, as its impure condition is caused in nearly every case by the introduction of bacteria of some kind, either pathogenic or non-pathogenic, and the long interval between milking the cow and delivering the milk to the consumer allows ample opportunity for the bacteria to multiply to such an extent that from one hundred thousand to a million can be found in one drop.

Milk, being the secretion of the mammary glands, is poured out, as a rule, only in the last stages of a pregnancy and after the birth. It is an opaque fluid, with a sweetish taste, and an odor recalling that of the animal from which it came. Its specific gravity varies from 1028 to 1035. Milk is an emulsion, consisting of water holding in solution casein, or cheese ; lactine, or sugar of milk; butter-fat, and ash—composed of sodium, potassium, chlorine, calcium, magnesium, phosphoric and sulphuric acid, and ferric oxide. At ordinary temperatures its alkaline reaction rapidly becomes acid, caused by the introduction of bacterium lactis, which converts the milk-sugar into lactic acid. The magnitude of the milk-business is astonishing, there being used in the United States $1,500,000,000 worth every year.

Veterinary inspection of dairies, dairy-cows, and milk as sold

[1] Paper read before the Pennsylvania State Veterinary Medical Society, September 21, 1897.

from milk-depots and vendors' wagons is a matter of vital importance to every consumer of the most necessary of all food-products that enter the human stomach. Every milk-inspector who thoroughly understands his business can do more to prolong the lives of the people living in his city or town than any other professional man, and he can, furthermore, check more cases of infantile digestive disturbance and disease than all the rest of the inhabitants in his community. There is absolutely no doubt in my mind that the veterinary milk-inspector can greatly prolong the average length of life by the proper exercise of extraordinary care of milk in all its relations. A large proportion of the deaths of people who die nowadays before reaching what is commonly called old age can be traced to the fact that microbes of every kind are allowed by them to enter their bodies through the swallowing of microbe-laden water, milk, or food, and through the inspiration of microbe-laden air. How many people are really careful to-day about the water or milk they drink, the food they swallow, or the air they breathe? And how many millions would be careful if they knew the difference between microbe-laden food and air and those which are pure?

Master minds all over the earth who are devoting their time, money, and energies to the invention and construction of terrible machines to kill other human beings, should devote their talents and means to the destruction of various bacteria that are more destructive and surer of accomplishing their purpose, and with more deadly effect than all the combined battle-machines ever invented by man. I refer to the perils that are all about us, that threaten us in the air we breathe, the water and milk we drink, and the food we eat. It is a sad and shameful fact that beings in the highest state of mental development should be absolutely at the mercy of billions and billions of unseen foes; that we should not know whether a mouthful of food or drink may bring us increased life and joy, or pain and death. Of all our food-products milk is the most easily affected by the myriads of bacteria that are constantly floating about us, and at all times, as milk is consumed largely by infants and invalids, and there are few homes where milk is not used in some form at every meal, by nearly every person, and for some it is an exclusive diet. Its composition makes it one of the very best mediums for bacteria to develop in. During the past ten years great strides have been made and much evidence has accumulated as to the causation of disease by milk; many cases of illness, and many epidemics are caused by the contaminated article.

Milk as it exists in the udder of a healthy cow is germ-free and pure. Milk is the first article of food for all mammalian life, and is more generally used than any other article of food, and is more easily contaminated than anything we eat; first, because of its wonderful absorbent powers and delicateness; second, because it is a most excellent medium for germs or bacteria to develop and multiply in, and the opportunity for the introduction of germs is greater than in any other article of diet that enters the human stomach, due to the fact that it is usually taken uncooked. It is a delicate fluid, rapidly decomposes, and requires constant vigilance to keep it of a high quality, and at the present time is produced with entirely too much carelessness, ignorance, and filth. Dirt in the form of dust is its most fruitful source of contamination.

It is a very unfortunate fact that milk must be drawn from a hair-covered udder on the under side of a hair-covered cow, as this part of a cow's hide retains particles of any dirt on which the cow may lie. The droppings of dust and bacteria from the hide of cows during milking, even when the animals are kept fairly clean, are very great. The introduction of dust from movements about the stables, caused by the shaking up of the bedding, the giving and eating of hay, are frightful causes of impure milk; and the bodily excretions also exert a very great influence in rendering milk impure.

The cleanliness of the hands and finger-nails of the milker are of vital importance. The hands should be dry; he who wets his fingers to milk is an unclean milker, as he or she, as it may be, in a mild manner washes the fingers in the milk, and some drops will fall into the pail. The outer garments of the milker must have attention, and should be clean when milking, as the different kinds of dust that necessarily gather about the clothing from being in the hay-mow, from cleaning horses and cattle, or various other sources, are not permissible in the milk-pails or other utensils. Carious molar teeth and a bad breath are incompatible with good milk; wounds and sores, when exposed, must be kept away from all milk-vessels, especially if of a syphilitic nature. To aid in the purity of milk each cow's udder and teats should be washed off with a clean cloth or sponge before every milking, and dried; if a sponge is used it should be scalded daily. The milk-house should be entirely separate from the dwelling and the stable; no family should live in or over a milk-house.

A really good article of milk or cheese cannot be produced in the cellar, kitchen, or other room of a dwelling-house unless specially separate from the dwelling apartments, and especially fitted for the care of milk, and kept as clean as soap and scalding-water and willing hands can make it. The milk-house or room must not be used as a wash-house or laundry. No barn-yard, pig-pen, or privy should be located near it. There has never yet been a milk-house kept too clean. Wooden vessels, because difficult to keep clean, should not be used, stone and glassware being better; but for convenience and economy tin is best; the glazing on stone and earthenware vessels cracks or checks, and it is hard to cleanse them. Soap, soda, moderately warm water and then scalding-water are the only proper things to use in cleansing milk-utensils.

At once, after the milk is removed from the cow, it should be strained and thoroughly cooled, and on this very point depends the keeping properties of a vast amount of milk that is properly handled every way except in cooling, and it is not cooled quickly enough nor cold enough; milk should never be allowed to be over 50° F. after milking; after being rapidly and thoroughly cooled, it should then be placed in a cold room, which must be kept cold, but not cold enough to freeze. Much depends on straining milk; the common wire-strainer is of no use from a pure-milk standpoint; the milk should be strained through several thicknesses of canton-flannel with the rough side of the cloth up. Milk must be kept free from all odors.

No chemicals should be used in preserving milk, as they are injurious and dangerous. The keeping power of milk depends on how rapidly and cleanly the cows are milked, how perfectly the milk is strained and cooled by the extraction of animal heat, and the complete removal from any kind of foul air.

Every moment milk remains exposed to the air it deteriorates. The common hay-bacillus, found in every barn, multiplies so fast that in twelve hours the descendants of two or three will number as high as 10,000,000,000, and they destroy the fat-globules and milk-sugar in a few hours. The only absolutely pure milk is sterilized milk, and this is just as easily contaminated when opened and not used at once.

The heavy mortality among bottle-fed babies is due to the character of the milk they have to digest. The medical profession has sought for a long time to remedy this evil, and as a result the market is filled with various artificial foods, all with claims of superiority; many are preferable to cow's milk when it is subject to the various impurities I have mentioned; but they would not have a shadow of a claim could the cow's milk be pure.

Alterations in milk arise from other than atmospheric and unsanitary conditions. Tainted food and water play their part, as well as many forms of disease—disturbance of the digestive tract from excessive, tainted, or insufficient food; diseases of the udder, bowels, blood; medicines, a few of which I enumerate: The various salts of iron, zinc, mercury, iodine, and turpentine, belladonna, aloes, arsenic, carbolic and salicylic acids, colchicum, salts of soda, camphor, hellebore, and many more that you are all as well acquainted with as with those I name. It would not be an unwise plan for our sister profession to treat many diseases of children by giving them medicinal remedies through cow's milk. The following vegetable matters exert a most unfavorable influence on milk: Thistles, garlic, onions, turnips, cabbage, dandelion, chestnut-tree leaves, distillery malt or brewers' grains, and last, but not least, ensilage. The preservation of green fodder and grains by stowing them in a close chamber, and tramping or pressing them down by weights, and keeping the mass air-tight causes fermentation, during which alcohol in a small quantity is formed, as well as acetic, lactic and enteric acids, and decomposition rapidly commences when exposed to the air. It is certainly an unsafe food, and, I believe, I was the first to condemn its use from a pure-milk standpoint, and I succeeded in having our city board of health refuse to grant a license to any vendor who uses ensilage for his cows. And so far as brewery and distillery swills and grains are concerned, no person who has proper knowledge can deny that the fermentation and chemical change that take place in brewers' grain by the transformation of the starch into sugar, and sugar into alcohol, which is the necessary part for the brewer's production, and the consequent removal of this part of the food, lessens its value as a food, especially for milk-cows. The process of fermentation produced by the change mentioned leaves the mass in such a condition that exposure for only a short time to atmospheric influences starts a fresh fermentation which renders the food unfit to feed milk-cows, whose milk is sold to supply general trade, composed of old and young people, to say nothing of the sick, who depend largely on a milk diet, and must have a suitable and nutritious food to meet the various demands of the system.

Diseases conveyed by milk may be divided into three classes: 1.

Those in which the germs are introduced into the milk by being conveyed from the body of the cow: Foot- and mouth-disease, anthrax, tuberculosis, and acute enteritis. 2. Those in which the germs are introduced into the milk from outside sources, like typhoid and scarlet fevers, diphtheria, and cholera. 3. That caused by a poison developed in the milk, called tyrotoxicon—a poisonous ptomaïn from bacterial growth.

Tuberculosis is the most important disease for our profession to deal with, and this fact makes it all the more necessary that all persons should know that tuberculosis has followed the introduction of milk-cows into every nation or country that they have entered; and, furthermore, that the number of cases and death-rate of consumption have increased in direct ratio with the increase of cattle. Tuberculosis exists in every type and breed of cattle, and finds its easiest victims among those kept specially for milk-purposes. The homes of the wealthiest in the world, and the homes of the poorest, testify that our meat- and milk-supply cause thousands of deaths from this disease every day. Consumption is no respecter of wealth, and it is the cause of one-seventh of all deaths. If all the victims of consumption who die annually lived in one country, newspapers would be crowded with stories about the most dreadful pestilence that ever visited the earth. In regard to the annual mortality among men from consumption I wish to mention the number who die among some of the insured orders. We will take the Royal Arcanum assessments for May, June, and July of this year, 1897. I find, out of 157 deaths in this order for May, that 18 died from tuberculosis. For the month of June there were 123 deaths, and 19 of them caused by tuberculosis. For the month of July 120 deaths, and 14 of them from tuberculosis. Now, these deaths, gentlemen, are among what were supposed to be healthy men, and, as we would say, were examined as to soundness when initiated in the order. The question now arises, had they latent tuberculosis when examined, or how many contracted the disease after examination?

Contamination of milk from impure water is of frequent occurrence, for such water is used in washing milk-utensils. A milk-dish should never be washed with the ordinary dish-rag, nor dried on the dish-cloth, but there should be separate cloths for washing and drying. Milk is frequently contaminated by submersion of the milk-can or pail in unclean water for cooling. The quality of the water used for the cleansing and cooling of milk-cans and pails has received too little attention. Fecal contamination of milk during the process of milking is great.

Epidemics due to impure and unhealthful milk have certain characteristics. The peculiarities of these epidemics, whether they be gastro-enteritis, scarlatina, typhoid fever, or diphtheria are, first, that the houses in which the people are affected are scattered; second, the cases appear very suddenly, many cases each day; third, the milk-drinkers are more liable to become diseased; fourth, houses of the wealthy are more apt to become seriously invaded than those of the poor, because more milk is taken. One-half of the epidemics due to milk-contamination can be traced to sickness among milk-handlers or members of their families. That foot- and mouth-diseases can be communicated we have numerous well-marked instances; in the epidemic of 1884, that occurred in Dover, England, there were 125 cases from one dairy; very fortunately, we are practically free from this malady. The cases of acute enteritis and tuberculosis are legion; I know of two well-marked cases in my city from the use of milk from a tuberculous cow that I traced beyond the possibility of a doubt. In this city of Franklin, I am told there were a number of cases of typhoid fever traced to the milk from a dairyman's home. During the Montclair, N. J., epidemic 135 cases out of a total of 150 were among the drinkers of milk from a filthy dairy where the owner had had typhoid fever. In our neighboring city of Titusville they had a serious epidemic of diphtheria caused by a dairyman's milk containing the bacillus. We have positive evidence of scarlet fever being communicated, for, all that, its bacilli has not been recognized, if such it has, and have almost daily reports of poisoning by milk and its products from tyrotoxicon.

We find, as a review of this subject, that the cow-stables should be so constructed that there be not less than 1500 cubic feet of air space per head. The stable must be thoroughly ventilated. The windows must be large, numerous and movable. The floors should be of cement or vitrified brick; there should be sewerage from each stall. Every manger should be separate. Cattle should not stand in rows facing each other. There should be a separate room in which to do the milking, and a separate apartment in which the cattle must be cleaned, and this room should be at the farthest point from feed-bins and milk-room. Every cow when milked should be covered with a thin sheet, made so as to envelop chest, abdomen, and posterior extremities; the same attendant should milk the same cows, and the milker should be free from disease.

Every milkman should understand how to select good dairy cows, and he should have a fair sample of the cow's milk tested as to quality before purchasing. A cow need not be necessarily high-bred to be a good dairy cow. Pure-bred Jerseys are more dangerous from a tuberculous standpoint than any other breed. Only healthy cows should be used in a dairy, and every one should be subjected to the tuberculin test. Only healthy people should be employed in the stables and to care for the milk and its receptacles; special attention to the cleanliness of the clothing, hands, and finger-nails of all people connected with the milk production; no visiting of sick people by the dairy hands. The dairy buildings should be not less than one hundred feet from any building, and should be constructed on higher ground and built so as to have a separate water supply of the purest water; the milk-house should be constructed so that it can be most thoroughly cleaned and be free from cracks, crevices, and all sources of dust of every character. The use of long troughs to feed and water cattle should be abolished; the old-fashioned milk-ticket that may be in one house this week and in another next week, must be stopped. The present plan of drawing milk from a can with a tin measure which is exposed to all sorts of atmospheric influences should not be allowed.

The quality of the milk is controlled by the quality and quantity of the feed. The dairy cow should always be fed ground grain. I advise the spaying of every dairy cow for the following reasons: She will give milk of a better quality for several years, the quality of the milk does not change as it does from monthly œstrum and subsequent stages of gestation and parturition and its sequels. All milk should be graded and sold according to quality, which would, in my estimation, tend to check adulteration to a very great extent. It is hardly fair that a vendor selling a superior quality of milk from healthy cows kept in the best possible condition, so far as stable sanitation and best quality of food are concerned, should only be able to get the same price that the filthy vendor with diseased and unthrifty cows can obtain. The variation of milk from the different breeds as well as the variation in the quality of morning and evening milk from the same cow of any breed make the necessity of graded milk and price a matter of great import to the vendor as well as the consumer.

(To be continued.)

---

The New York College of Veterinary Surgeons has some thirty students this year, among them one young woman.

